# Altered Behavioral Responses Show GABA Sensitivity in Muscleblind-Like 2-Deficient Mice: Implications for CNS Symptoms in Myotonic Dystrophy

**DOI:** 10.1523/ENEURO.0218-22.2022

**Published:** 2022-10-07

**Authors:** Kamyra S. Edokpolor, Anwesha Banerjee, Zachary T. McEachin, Jingsheng Gu, Adam Kosti, Juan D. Arboleda, Paul S. García, Eric T. Wang, Gary J. Bassell

**Affiliations:** 1 Department of Cell Biology, Emory University School of Medicine, Atlanta, GA 30322; 2 Department of Human Genetics, Emory University School of Medicine, Atlanta, GA 30322; 3 Department of Anesthesiology, Columbia University, New York, NY 10032; 4 Department of Molecular Genetics and Microbiology, Center for Neurogenetics, University of Florida, Gainesville, FL 32610

**Keywords:** anesthesia, benzodiazepine, GABA, hypersomnia, Mbnl2, Myotonic Dystrophy Type 1

## Abstract

Considerable evidence from mouse models and human postmortem brain suggests loss of Muscleblind-like protein 2 (MBNL2) function in brain is a major driver of CNS symptoms in Myotonic dystrophy type 1 (DM1). Increased hypersomnia, fatigue, and surgical complications associated with general anesthesia suggest possible sensitivity to GABAergic inhibition in DM1. To test the hypothesis that MBNL2 depletion leads to behavioral sensitivity to GABA_A_ receptor (GABA_A_-R) modulation, *Mbnl2* knock-out (KO) and wild-type (WT) littermates were treated with the anesthetic sevoflurane, the benzodiazepine diazepam, the imidazopyridine zolpidem, and the benzodiazepine rescue agent, flumazenil (Ro 15-1788), and assessed for various behavioral metrics. *Mbnl2* KO mice exhibited delayed recovery following sevoflurane, delayed emergence and recovery from zolpidem, and enhanced sleep time at baseline that was modulated by flumazenil. A significantly higher proportion of *Mbnl2* KO mice also loss their righting reflex [loss of righting reflex (LORR)] from a standard diazepam dose. We further examined whether MBNL2 depletion affects total GABA_A_-R mRNA subunit levels and validated RNA-sequencing data of mis-spliced *Gabrg2*, whose isoform ratios are known to regulate GABA sensitivity and associated behaviors. While no other GABA_A_-R subunit mRNA levels tested were altered in *Mbnl2* KO mouse prefrontal cortex, *Gabrg2S/L* mRNA ratio levels were significantly altered. Taken together, our findings indicate that loss of MBNL2 function affects GABAergic function in a mouse model of myotonic dystrophy (DM1).

## Significance Statement

CNS symptoms in Myotonic dystrophy type 1 (DM1) could be partly driven by excess GABAergic inhibition. DM1 patients experience high rates of fatigue and hypersomnia together with postoperative complications in response to anesthetics. The behavioral neuropharmacology data shown here implicate loss of MBNL2 as a driver of GABA sensitivity in DM1. Furthermore, since *Mbnl2* knock-out (KO) mice phenocopy CNS symptoms of DM1 and recapitulate numerous mis-splicing events observed in human brain, the present study highlights one potential RNA misprocessing event that may contribute to these clinical symptoms.

## Introduction

CNS symptoms such as fatigue, hypersomnia, excessive daytime sleepiness, and adverse responses to anesthesia are often debilitating or, in the case of anesthesia, potentially fatal, for individuals with myotonic dystrophy (Aldridge, 1985; Speedy, 1990; Butler et al., 2000; Heatwole et al., 2014; Laberge et al., 2020; Miller et al., 2021). General anesthetics, such as isoflurane, sevoflurane and propofol, potentiate extrasynaptic GABA_A_ receptor (GABA_A_-R), which mediate tonic, inhibitory currents (Garcia et al., 2010). Interestingly, prolonged and heightened sensitivity to analgesics and sedatives cause reduced levels of consciousness, exaggerated ventilatory weakness, pharyngeal dysfunction, and gastrointestinal dysmotility during recovery in Myotonic dystrophy type 1 (DM1) patients (Ferschl, 2016). It should be noted that both hypersomnia and excessive daytime sleepiness (Subramony et al., 2020) exacerbate delayed anesthetic recovery and are also associated with cognitive dysfunction such as memory deficits and delirium (Safavynia et al., 2016). An early clinical study also noted that DM patients exhibit enhanced sensitivity to barbiturates and benzodiazepines, which enhance the activity of GABA_A_-R (Harper, 2001). Taken together, these findings suggest that there may be dysregulated responses of GABA_A_-R in DM1 that may underlie these CNS symptoms.

DM1 is a multisystemic, autosomal dominant disease caused by microsatellite CTG expansions in the 3′ untranslated region (UTR) of the *DMPK* gene (Malik et al., 2021). Transcription of these repeats generate CUG containing RNAs that form toxic intranuclear RNA foci that sequester MBNL RNA binding proteins. While MBNL proteins play critical roles in various steps of RNA processing and localization (Wang et al., 2012; Batra et al., 2014; Taliaferro et al., 2016), their role in alternative splicing has been shown to be relevant to DM1 pathophysiology. Sequestration of MBNLs within the nucleus results in the presence of fetal splicing patterns in adult tissues (Wang et al., 2015). Some of these aberrant splicing patterns are linked to phenotypes in muscle and heart. For example, inclusion of chloride channel 1 exon 7a causes myotonia (Wheeler et al., 2007), skipping of Bin1 exon 11 causes muscle wasting and weakness (Fugier et al., 2011), and aberrant usage of sodium channel SCN5A exon 5A causes cardiac conduction defects (Freyermuth et al., 2016). However, no MBNL-dependent splicing changes have been functionally linked to phenotypes in the CNS.

The *Mbnl2* knock-out (KO) mouse has been a critical animal model to investigate impairments in neurologic function that correlate with numerous mis-splicing events in brain, where MBNL2 is predominantly expressed (Chen et al., 2018). *Mbnl2* KO mice show decreased synaptic NMDA receptor activity, impaired long-term potentiation (LTP), deficits in hippocampal-dependent learning/memory, and increased REM sleep episodes (Charizanis et al., 2012). While *Mbnl2* KO mice show hippocampal-dependent deficits, of relevance to the present study, we focus on the cortex region as it is heavily involved in sleep and anesthesia (Hudetz, 2012). Additionally, transcriptomic studies have identified common alternative splicing events in the cortex of *Mbnl2* KO mouse and DM1 postmortem brain (Goodwin et al., 2015; Otero et al., 2021). *Mbnl2* KO mice or sequestration of MBNL2 in human DM1 results in mis-splicing of *Gabrg2* (Charizanis et al., 2012; Weyn-Vanhentenryck et al., 2018; Otero et al., 2021) in which exon 9 is excluded, producing the short, fetal isoform (*Gabrg2S*). Although *Gabrg2* is only one candidate identified among others that could impact the GABA axis in DM1, the γ2 subunit is a component of almost 60% of all GABA_A_-R.

Here, we use behavioral neuropharmacology methods to test the hypothesis that MBNL2 depletion affects GABA sensitivity. We demonstrate that the *Mbnl2* KO mouse model of DM1 exhibits behavioral sensitivity to anesthesia, benzodiazepines, and GABA_A_-R modulation. We further provide an independent experimental validation of *Gabrg2* mis-splicing observed previously from transcriptome wide analyses (Charizanis et al., 2012; Weyn-Vanhentenryck et al., 2018).

## Materials and Methods

### Animals

*Mbnl2* KO mice maintained on a hybrid background of C57BL/6 and 129S strains were provided from the laboratory of Maurice Swanson at the University of Florida-Gainesville (Charizanis et al., 2012), and subsequently bred and group housed at Emory University animal facilities. Mice used in experiments were backcrossed twice with the following mating scheme: *Mbnl2*^+/−^ x 129S^+/+^ > Mbnl2/129S^+/−^ x C57BL/6^+/+^ > offspring^+/−^ x 129S^+/+^ > offspring^+/−^ x C57BL/6^+/+^ > offspring^+/−^ x offspring ^+/−^. The animal protocol was approved by the Institutional Animal Care and Use Committees of Emory University and complied with the Guide for the Care and Use of Laboratory Animals. Mice were housed on a 12/12 h light/dark cycle with access to standard mouse chow and water *ad libitum*. Four- to five-month-old, naive male and female mice were used once for each behavioral experiment.

### RT-qPCR

For RNA extraction from prefrontal cortex mouse tissue, ∼30 mg of tissue was homogenized in a lysis buffer (TRIzol) using a bullet blender tissue homogenizer (Next Advance). RNA lysates were cleared by spinning samples at 10,000 × *g* for 1 min. Cleared lysates were used for RNA extraction as per the manufacturer’s protocol. cDNA was obtained via RT-PCR using the High-Capacity cDNA Reverse Transcription kit (Thermo Fisher Scientific). To quantify relative mRNA expression of *Gabrg2S and Gabrg2L*, qPCR was performed for each sample using custom TaqMan gene expression assays (Thermo Fisher Scientific) on a Quantstudio 6 Flex system (Applied Biosystems). The following custom designed FAM-labeled TaqMan assays were used: *Gabrg2S* (*Exon 8-Exon 10*): APZTHKZ, and *Gabrg2L* (*Exon 9-Exon 10*): APYMNZ3. For *Gabaa* receptor qPCR analysis of tissue samples, the following FAM-labeled TaqMan assays were used: *Gabra1* (Mm00439044), *Gabra2* (Mm01294271), *Gabra3* (Mm01294271), *Gabra4* (Mm00802631), *Gabra5* (Mm00621092), *Gabrad* (Mm01266203), *Gabrb1* (Hs00181306), *Gabrb3* (Mm00433473), *Gabrg1* (Mm00439047), *Gabrg2* (Mm01227748), and *Gabrg3* (Mm00433494). Relative tissue RNA expression was normalized to *Gapdh*.

### RNA fragment analysis

RT-PCR samples were created using following forward and reverse primer sequences for *Gabrg2L* (FWD*: ggcaccctgcattattttgt*, REV: *ttgaaggtgtgtggcattgt*) then visualized and analyzed using an Advanced Analytical Agilent Fragment Analyzer system. This system uses capillary electrophoresis and an intercalating dye for fragment separation, respectively. Samples were prepared and run on the Fragment Analyzer system using the dsDNA 905 reagent kit (DNF-905-KO500). Relative fluorescent units (RFU) were then used to determine relative abundance of fragments found in each sample. Percent Spliced In (PSI) was calculated by using the RFU values of the fragments of interest for each sample and determining the ratio of *Gabgr2L* abundance to the combined abundance of both *Gabrg2S* and *Gabrg2L* in each sample.

### Anesthesia experimental design (emergence and recovery behavioral markers)

Before induction of general anesthesia, mice were tested for successful removal of adhesive tape applied to paw three times to ensure no baseline deficit in muscular ability. For induction, mice were placed in a prefilled anesthesia induction chamber of 6% sevoflurane (Patterson Veterinary). Mice were considered “anesthetized” when they were unable to right themselves [loss of righting reflex (LORR)], after being placed on their back. The righting reflex is common in prey animals that do not sleep supine and is determined to be absent when animal has received sufficient analgo-sedative agents to prevent their automatic transition from supine to all four paws beneath the body (on the surface of the chamber; Bignall, 1974; Jusufi et al., 2011). Immediately after LORR, mice were placed supine on a heating pad adjusted to maintain body temperature between 38.4–39°C. The gas flow was switched from the induction chamber to the anesthetic nose cone placed over the mouse’s nose. Anesthesia was maintained for 60 min (3.6% in 1 l/min oxygen). Body temperature, respiratory rate, heart rate, oxygen saturation measured by pulse oximetry (SpO2), and expired gases were measured at 5-min intervals. After 60 min, the anesthetic was switched off and mice were allowed to passively emerge from anesthesia. The time to return of righting reflex (RORR) and adhesive tape removal around the paw was measured as indicators of emergence and recovery from anesthesia, respectively. Researcher was blinded for all tests.

### Benzodiazepine and imidazopyridine administration

To determine effects of diazepam or zolpidem on *Mbnl2* KO and wild-type (WT) littermates, diazepam (Alomone Labs) was injected intraperitoneally at a dose of 60 mg/kg (6 mg/ml in 0.2% Tween 20/0.9% sterile saline). Zolpidem (Alomone Labs) was injected intraperitoneally at a dose of 60 mg/kg (6 mg/ml in 0.9% sterile saline; Kralic et al., 2002). Mice were then placed in a warmed home cage (without bedding) and behaviors recorded using a video camera. Videos were later scored by researchers that were blinded to experimental conditions. Once placed in the cage, animals were tested for the loss of righting reflex (LORR) as previously described (Quinlan et al., 2000; Chandra et al., 2005; Han et al., 2019). The length of time from diazepam or zolpidem administration until onset of LORR was recorded as LORR latency. LORR duration was calculated by subtracting the time of onset of LORR from the time at which the animal regained the righting reflex.

### Sleep activity and flumazenil (Ro 15-1788) administration

Determining sleep immobility for each mice followed a modified protocol previously published (Fisher et al., 2012). Briefly, after acclimation period mice were placed in a square box (1.2 × 1.2 m) in a quiet room with food and water for 4 h duration (starting between 10 A.M. and 12 P.M.). Animals were tracked using an automated video tracking software (ANY-maze, Stoelting) to detect total distance traveled (meters) and time immobile/sleep episodes (seconds) of the mouse. Sleep was defined as the period when the mice was at least 95% immobile for a stretch of 40 s (Pack et al., 2007; Fisher et al., 2012; Heise et al., 2015; Pritchett et al., 2015; Pilorz et al., 2016; Hirano et al., 2018; Lee et al., 2018; Banks et al., 2020). Briefly, all animals were first acutely administered 30 mg/kg of vehicle via oral gavage (Labrasol, Gattefossé). Animals were staggered and only two mice were recorded each day to ensure we collect data during the same time of the day and avoid changes in sleep pattern because of circadian changes. Once the measurement of baseline activity was completed, all animals were treated with an acute dose of 30 mg/kg of flumazenil (Expansion Therapeutics). Sleep activity was measured in the same manner as described above. Animals were staggered in terms of receiving either vehicle or flumazenil such there was a gap of 3 d for each mice receiving oral gavage, thereby reducing sequential related stress bias.

### Statistical analyses

Behavioral experiments were performed in 3 or more independent cohorts and the data were pooled. Each molecular experiment was replicated at least 2 independent times. Normal distribution within all datasets was assessed using the Shapiro–Wilk’s test. The χ^2^ test, Welch’s *t* test, paired *t* test, three-way repeated measure ANOVA, and Spearson’s correlation were used for statistical analysis where indicated. Tukey’s and Sidak multiple comparisons test were used for ANOVA where noted. Datasets that did not display normal distribution were analyzed by the nonparametric Mann–Whitney. Significance threshold was set as *p *≤* *0.05. Two-way ANOVA tests were conducted between WT and M*bnl2* KO mice with sex as the cofactor and revealed no sex difference therefore, both male and females were combined for all experiments. All datasets are displayed as mean ± SEM. Statistical testing was performed using Prism 9.3 (GraphPad Software). Outliers determined and removed by ROUT outlier test for all experiments. Exclusion criteria is based on false discovery rate (FDR), where Q was set to 1%.

## Results

### Reduced *Gabrg2L* inclusion in Mbnl2 KO prefrontal cortex

**Figure 1.**
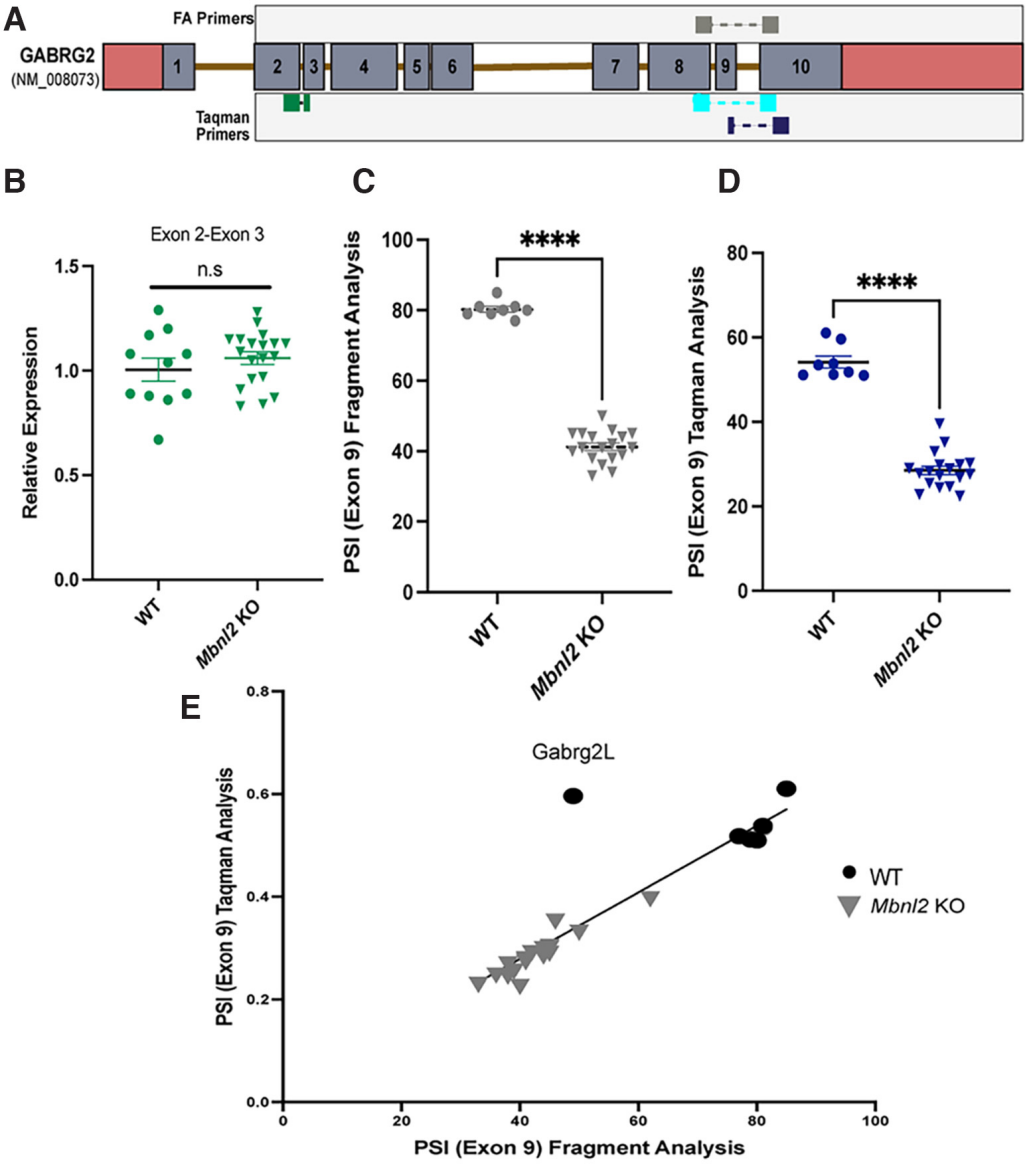
Reduced *Gabrg2L* inclusion in *Mbnl2* KO prefrontal cortex. ***A***, Schematic of custom TaqMan primers for *Gabrg2S* (Exon 8-Exon 10), *Gabrg2L* (Exon 8-Exon 9; Exon 9-Exon 10), and primers (Exon 8-Exon 10) for RNA fragment analysis. ***B***, Constitutively expressed Exon 2-Exon 3 of *Gabrg2* is not significantly different between genotypes (*t*_(0.99)_ = 15.33, *p *=* *0.36, Welch’s *t* test). ***C***, Significant reduction of *γ2L* inclusion in *Mbnl2* KO mice detected via RNA fragment analysis (*t*_(29.43)_ = 23.11, *p *<* *0.0001, *t*_(14.70)_ = 14.38, *p *<* *0.0001, Welch’s *t* test). ***D***, Significant reduction of *γ2L* inclusion in *Mbnl2* KO detected via Taqman assay (*t*_(14.70)_ = 14.38, *p *<* *0.0001, Welch’s *t* test). Error bars are ± SEM. ***E***, Strong, positive correlation between PSI values generated from RNA fragment analysis and custom TaqMan assay (*r*_(26)_ = 0.947, *p* ≤ 0.0001, Spearman’s correlation). Comprehensive mRNA analyses of *Gabaa-R* subunits in prefrontal cortex of *Mbnl2* KO mice revealed no other subunit difference (Extended Data Fig. 1-1). Full statistical analyses of both experiments can be viewed in Extended Data Figure 1-2.

We performed an independent analysis of *Gabrg2* mis-splicing observed previously from a transcriptome wide analysis in *Mbnl2* KO mice and human DM1 postmortem brain (Charizanis et al., 2012; Otero et al., 2021; Degener et al., 2022). We designed custom TaqMan primers and DNA primers for *Gabrg2S* and *Gabrg2L* to span exon junctions (Fig. 1*A*). To confirm total *Gabrg2* mRNA levels were no different in *Mbnl2* KO versus WT mice, we tested a non-MBNL2 regulated exon junction, exon 2-exon 3 (*t*_(0.99)_ = 15.33, *p *=* *0.360, Welch’s *t* test; Fig. 1*B*). We also conducted RNA fragment analysis of *Mbnl2* KO (*N* = 19) and WT (*N* = 11; Fig. 1*C*) to correlate with PSI values calculated from TaqMan analysis (Fig. 1*D*). Both methods revealed significant reduction of exon 9 inclusion in *Mbnl2* KO mice (*t*_(29.43)_ = 23.11, *p *<* *0.0001, *t*_(14.70)_ =14.38, *p *< 0.0001, Welch’s *t* test, respectively). The TaqMan PSI values were also strongly correlated with PSI values from fragment analysis (*r*_(26)_ = 0.947, *p* ≤ 0.0001, Spearman’s correlation; Fig. 1*E*). Assessment of other *Gaba_a_* receptor subunit mRNAs in prefrontal cortex samples of *Mbnl2* KO and WT mice, also by TaqMan qPCR, revealed no significant differences in steady state levels at three months of age (Extended Data Fig. 1-1). Full statistical analyses provided in Extended Data Figure 1-2.

10.1523/ENEURO.0218-22.2022.f1-1 Extended Data Figure 1-1 *Gabaa*-*R* mRNA analysis of prefrontal cortex WT and *Mbnl2* KO mice at three months of age. No significant differences detected in relative expression of majority of *Gabaa* mRNA receptors between *Mbnl2* KO and WT mice at three months of age. A two-way mixed effect analysis revealed a significant effect between *Gabaa*-*R* (*F*_(4.238,58.86)_ = 13.08, *p *<* *0.0001, ANOVA); however, upon Sidak’s multiple comparisons test, no significant difference was detected between WT and *Mbnl2* KO *Gabaa*-*R* mRNA levels (*N* = 7WT, 8 *Mbnl2* KO), except *Gabra1* (*N* = 11 WT, 19 *Mbnl2* KO). Error bars represent SEM. Download Figure 1-1, TIF file.

**Figure 2.**
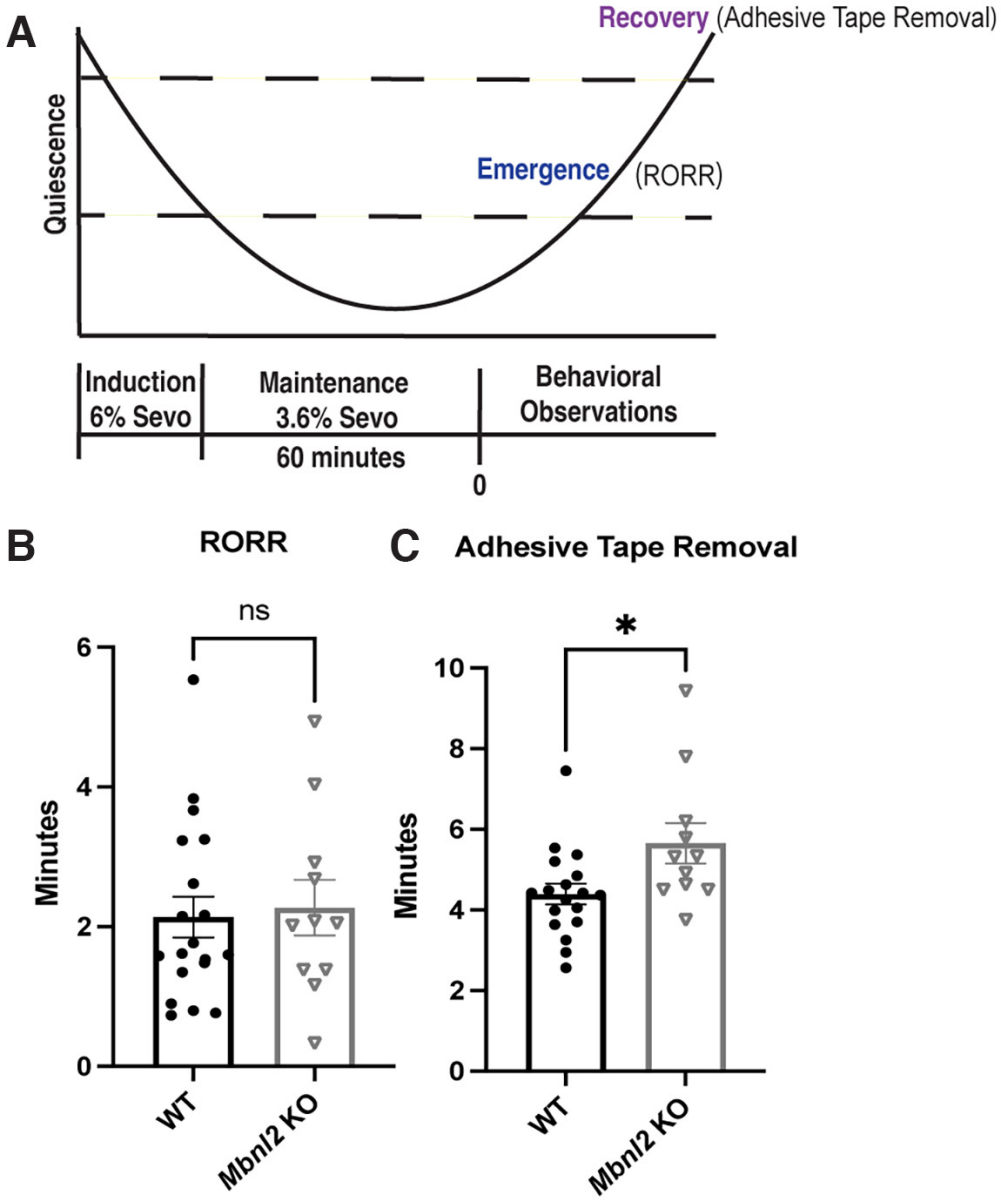
*Mbnl2* KO mice recover significantly slower from Sevoflurane administration. ***A***, Illustration of inhalant anesthetic procedure. ***B***, *Mbnl2* KO (*N* = 11) and WT (*N* = 19) mice displayed no significant difference in time to RORR (return of righting reflex; *t*_(20.31)_ = 0.274, *p *=* *0.79, Welch’s *t* test). ***C***, *Mbnl2* KO (*N* = 11) mice took significantly longer to recover compared with WT mice (*t*_(15.49)_ = 2.23, *p *=* *0.04, Welch’s *t* test). Error bars are ± SEM. No muscular deficits were detected in *Mbnl2* KO mice (Extended Data Fig. 2-1). Full statistical analyses of experiments can be viewed in Extended Data Figures 2-2 and 2-3, respectively.

10.1523/ENEURO.0218-22.2022.f1-2 Extended Data Figure 1-2 Statistical results from RT-qPCR and RNA fragment analyses. Download Figure 1-2, XLS file.

### *Mbnl2* KO mice recover significantly slower from sevoflurane administration

As MBNL2 is enriched in brain, whereas MBNL1 is abundant in muscle, the *Mbnl2* KO mouse model have been successfully used to study brain defects, altered sleep, and hippocampal function with no confounding motor deficits (Charizanis et al., 2012). We first confirmed no deficit in wire hang time or total distance traveled (open field test; Extended Data Figs. 2-1, 2-2).

Prolonged sensitivity to analgesics and sedatives are known to exacerbate delayed anesthetic recovery which is associated with cognitive dysfunction such as memory deficits and delirium (Song et al., 2018). Sevoflurane is a commonly used anesthetic that exerts its effects in part via the potentiation of extrasynaptic GABA_A_ receptors (Garcia et al., 2010). Owing to the adverse responses to anesthesia in DM1 (Butler et al., 2000), we hypothesized that *Mbnl2* KO mice would demonstrate difficulty recovering from sevoflurane. After 1 h of 3.6% sevoflurane exposure, *Mbnl2* KO (*N* = 12) mice did not demonstrate difficulty with emergence as determined by return of righting reflex (RORR; *t*_(20.31)_ = 0.274, *p *=* *0.786, Welch’s *t* test) compared with WT mice (*N* = 19; Fig. 2*B*). However, significant delays in recovery as detected via adhesive tape removal was found in *Mbnl2* KO mice (*t*_(15.49)_ = 2.23, *p *=* *0.040, Welch’s *t* test; Fig. 2*C*). Full statistical analyses provided in Extended Data Figure 2-3.

10.1523/ENEURO.0218-22.2022.f2-1 Extended Data Figure 2-1 No neuromuscular (Wire hang test) or locomotor deficits observed in *Mbnl2* KO mice. ***A***, Briefly, mice were placed on an elevated wire grid above which was then inverted and suspended above a cage; the latency to when the animal falls was recorded and averaged three times. No significant difference in hang time detected between genotypes (*t*_(1.073)_ = 35.57, *p *=* *0.55; Welch’s *t* test). ***B***, No significant difference in distance travelled between genotypes (*t*_(0.748)_ = 21.98, *p* = 0.46, Welch’s *t* test). Download Figure 2-1, TIF file.

10.1523/ENEURO.0218-22.2022.f2-2 Extended Data Figure 2-2 Statistical results from wire hang test and open field (distance travelled). Download Figure 2-2, XLS file.

**Figure 3.**
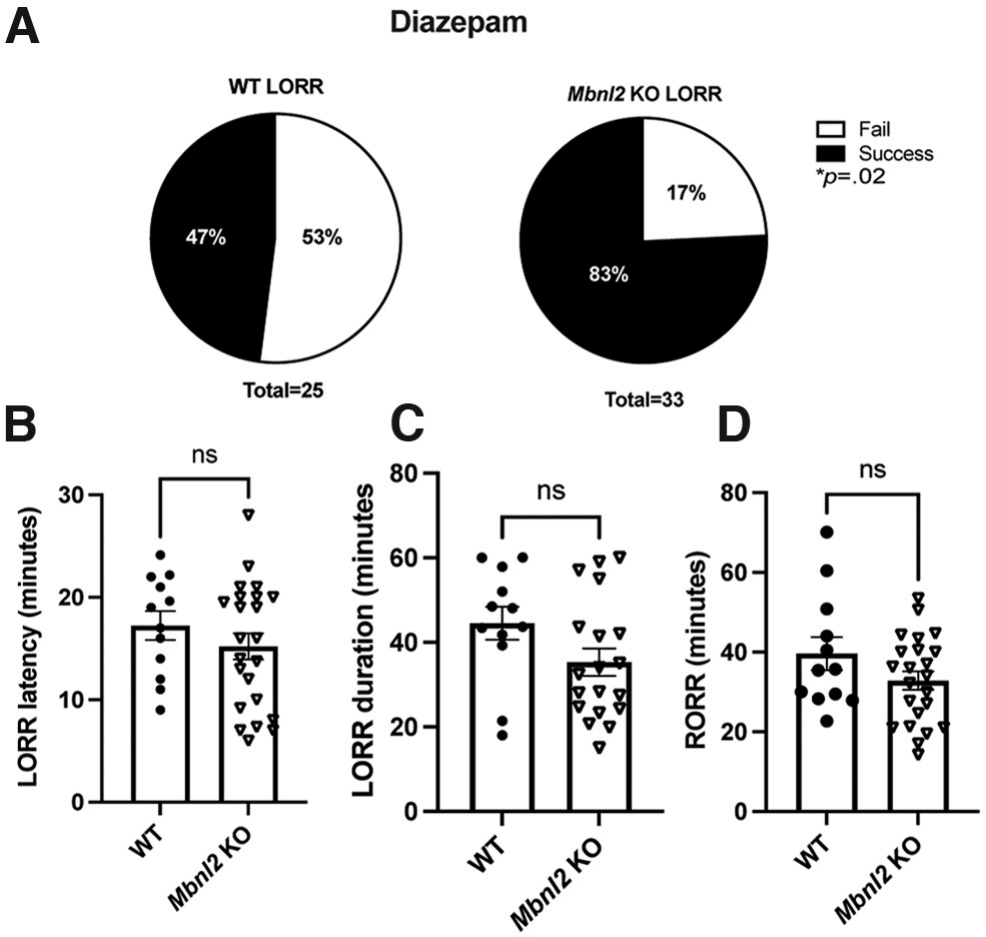
A significantly higher percentage of Mbnl2 KO mice exhibit (loss of righting reflex) LORR (lose their righting reflex) with diazepam. *Mbnl2* and WT mice administered 60 mg/kg of diazepam (intraperitoneal; ***A***) *Mbnl2* KO mice (*N* = 33) are significantly more likely to LORR compared with WT (*N* = 25) mice [*X*^2^ (1, *N* = 37) = 4.74, *p *=* *0.02]. ***B–D***, No significant difference in LORR metrics (LORR latency, RORR, LORR duration) was found between *Mbnl2* KO and WT littermates (*t*_(27.19)_ = 1.07, *p *=* *0.29, Welch’s *t* test; (*t*_(17.83)_ = 1.42, *p* = 0.17, Welch’s *t* test; *t*_(24.30)_ = 1.81, *p *=* *0.08, Welch’s *t* test). Error bars are ± SEM. Full statistical analyses can be viewed in Extended Data Figure 3-1.

10.1523/ENEURO.0218-22.2022.f2-3 Extended Data Figure 2-3 Statistical results from sevoflurane experiment. Download Figure 2-3, XLS file.

### A significantly higher percentage of *Mbnl2* KO mice LORR (lose their righting reflex) with diazepam

We next tested the pharmacological effect of the benzodiazepine, diazepam, on LORR behavioral metrics (LORR percentage, latency, duration, and return of righting.) It is well established that benzodiazepines bind at the interface of the ɑ and γ2 subunits, thus there is an obligatory role of the γ2 subunit for benzodiazepine activity (Schweizer, 2003). A significantly higher number of *Mbnl2* KO mice lost their righting reflex compared with their WT littermates after a clinically relevant dose of 60m/kg of diazepam for sedation [*X*^2^ (1, *N* = 37) =4.74, *p *=* *0.02, χ^2^; Fig. 3*A*]. Neither latency to LORR (*t*_(34)_ =1.07, *p *=* *0.29, Welch’s *t* test), RORR (*t*_(34)_ =1.07, *p* = 0.17, Welch’s *t* test) nor duration significantly differed between genotypes (*t*_(34)_ = 1.07, *p *=* *0.08, Welch’s *t* test; Fig. 3*B–D*). Full statistical analyses provided in Extended Data Figure 3-1.

10.1523/ENEURO.0218-22.2022.f3-1 Extended Data Figure 3-1 Statistical results from diazepam experiment. No significant difference in LORR metrics between WT and *Mbnl2* KO mice administered THIP. *Mbnl2* and WT mice were administered 30 mg/kg of THIP (intraperitoneal). ***A***, No propensity for *Mbnl2* KO (*N* = 6) mice to LORR at higher rate compared to WT [*N* = 8 mice; *X*^2^ (1, *N* = 23) = 1.245, *p* = 0.26]. ***B–D***, No significant difference in LORR latency, RORR, or LORR duration between genotypes (*t*_(0.949)_ = 6.588, *p* = 0.38, Welch’s *t* test; *t*_(0.793)_ = 11.03, *p* = 0. 0.39, Welch’s *t* test; *t*_(0.742)_ = 10.16, *p* = 0.48, Welch’s *t* test). Download Figure 3-1, XLS file.

### Slower RORR and longer LORR duration observed in *Mbnl2* KO mice administered zolpidem

**Figure 4.**
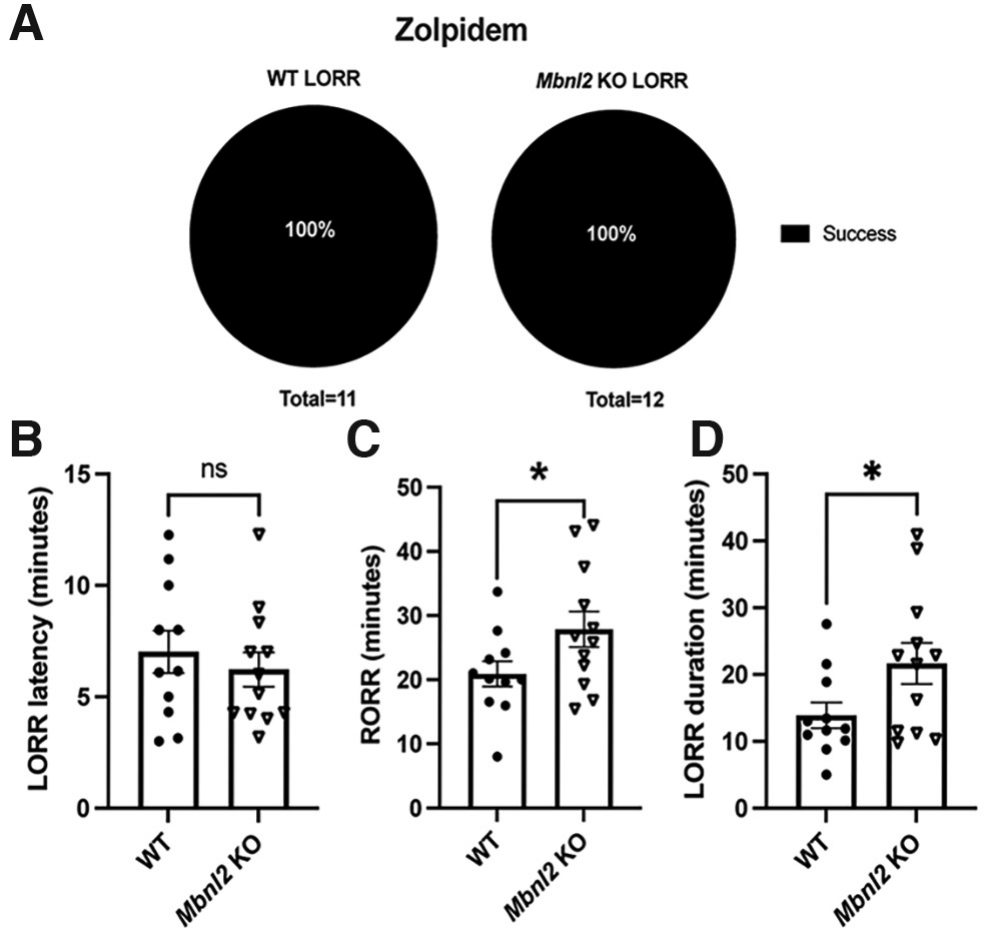
Slower RORR (return of righting reflex) and longer LORR duration in *Mbnl2* KO mice administered zolpidem. *Mbnl2* and WT mice were administered 60 mg/kg of zolpidem (intraperitoneal). ***A***, No propensity for *Mbnl2* KO (*N* = 12) mice to LORR at higher rate compared with WT (*N* = 11) mice as LORR rate was 100% successful for both genotypes. ***B***, No significant difference in LORR latency between genotypes (*t*_(19.62)_ = 0.646, *p* = 0.53, Welch’s *t* test. ***C***, *Mbnl2* KO mice take significantly longer to emerge than WT mice (RORR; *t*_(2.03)_ = 19.60, *p* = 0.05, Welch’s *t* test). ***D***, *Mbnl2* KO mice display longer sedation period (LORR duration) compared with WT mice (*t*_(2.133)_ = 18.24, *p* = 0.05, Welch’s *t* test.) No significant difference in LORR metrics between WT and *Mbnl2* KO mice administered THIP (Extended Data Fig. 4-1). Error bars are ± SEM. Full statistical analyses provided in Extended Data Figures 4-2 and 4-3.

We next tested zolpidem, which is specific to the γ2 and α1 subunit interface (Hanson et al., 2008). Using a supratherapeutic dose of 60 mg/kg (Bohnsack et al., 2018), neither LORR percentage (Fig. 4*A*), nor latency (*t*_(19.62)_ = 0.646, *p *=* *0.525, Welch’s *t* test) were significantly different (Fig. 4*B*); however, longer LORR duration (*t*_(2.133)_ =18.24, *p *=* *0.046, Welch’s *t* test; Fig. 4*C*) and slower RORR (*t*_(2.03)_ = 19.60, *p *=* *0.051, Welch’s *t* test; Fig. 4*D*) were detected in *Mbnl2* KO mice compared with WT littermates. Full statistical analyses provided in Extended Data Figure 4-2. 4,5,6,7-tetrahydroisoxazolo (5,4-*c*)pyridin-3(-ol) (THIP) is a selective ligand for extrasynaptic GABA_A_-R. THIP specifically targets δ-subunit containing GABA_A_-R receptors which are known to pair with the GABA_A_-α4-R subunits (Belelli et al., 2005; Drasbek and Jensen, 2006; Winsky-Sommerer et al., 2007). Because THIP has been found to be a less sensitive ligand with extrasynaptic γ2 subunit expression (Meera et al., 2011), we wanted to determine whether THIP had any effect on *Mbnl2* KO mice. No significant difference between WT and *Mbnl2* KO mice was found on any LORR metric following an intraperitoneal injection of 30 mg/kg of THIP (Extended Data Fig. 4-1; statistical analyses in Extended Data Fig. 4-3).

10.1523/ENEURO.0218-22.2022.f4-1 Extended Data Figure 4-1 No significant difference in LORR metrics between WT and *Mbnl2* KO mice administered THIP. *Mbnl2* and WT mice were administered 30mg/kg of THIP (IP). (A) No propensity for *Mbnl2* KO (N= 6) mice to LORR at higher rate compared to WT (N=8 mice) (*X*^2^ (1, N=23) = 1.245, *p*= 0.26). (B-D) No significant difference in LORR latency, RORR, or LORR duration between genotypes (*t*(0.949)= 6.588, *p* = 0.38, Welch’s t-test; (*t*(0.793)= 11.03), *p* = 0. 0.39, Welch’s’ t-test, (*t*(0.742)=10.16, p= 0.48. Welch s t-test.) Download Figure 4-1, TIF file.

10.1523/ENEURO.0218-22.2022.f4-2 Extended Data Figure 4-2 Statistical results from zolpidem experiment. Download Figure 4-2, XLS file.

10.1523/ENEURO.0218-22.2022.f4-3 Extended Data Figure 4-3 Statistical results from THIP experiment. Download Figure 4-3, XLS file.

### Sleep behavior in *Mbnl2* KO mice is selectively and transiently modulated by flumazenil (Ro 15-1788)

**Figure 5.**
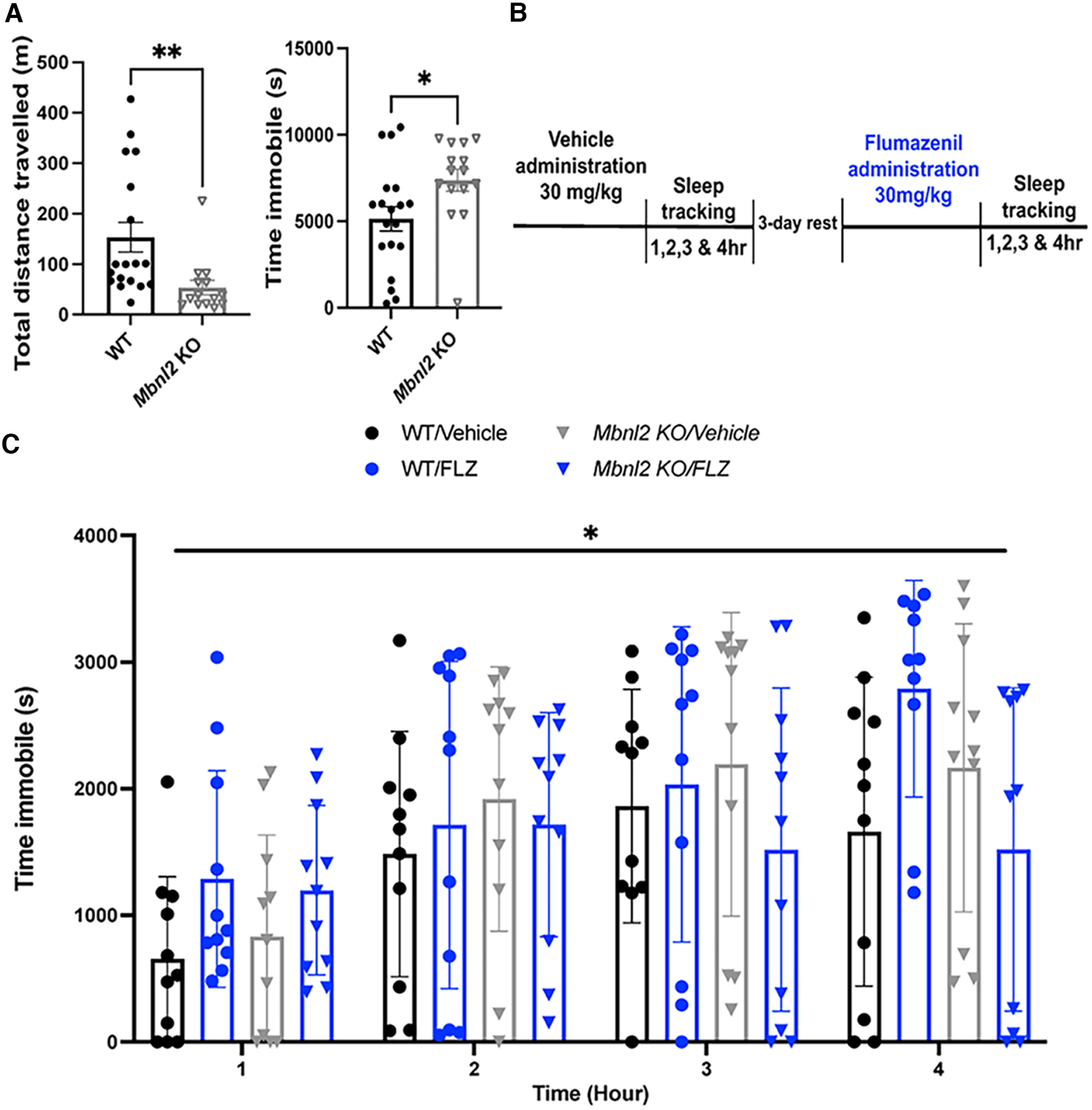
Altered sleep behavior in *Mbnl2* KO mice is selectively modulated with flumazenil (Ro 15-1788). ***A***, *Mbnl2* KO mice (*N* = 13) travel significantly less (*t*_(3.02)_ = 24.36, *p *=* *0.005, Welch’s *t* test) and spend a significantly longer time immobile than WT littermates (*N* = 18; *t*_(2.36)_ = 31.99, *p *=* *0.02, Welch’s *t* test). ***B***, Schematic of within-subject design experiment of flumazenil (Ro 15-1788; *Ro 15-1788*) versus vehicle by oral gavage. Time immobile was analyzed at each hour posttreatment. ***C***, Significant effect of time on total time immobile (*F*_(3,30)_ = 13.14, *p *<* *0.0001, three-way ANOVA; *N* = 10) and significant interaction between flumazenil (Ro 15-1788; *Ro 15-1788*) 30 mg/kg treatment and genotype on total time immobile (*F*_(1,10)_ = 6.398, *p *=* *0.03, three-way ANOVA) in *Mbnl2* KO mice (*N* = 10). Error bars are ± SEM. Flumazenil (Ro 15-1788) decreased time immobile in *Mbnl2* KO mice versus WT mice (Extended Data Fig. 5-1). Full statistical analyses are provided in Extended Data Figure 5-1. Individual plots of WT and *Mbnl2* KO mice response to flumazenil (Ro 15-1788) administration are shown in Extended Data Figure 5-2. RNA fragment analyses of *Gabrg2L* inclusion in mice from experiment are shown in Extended Data Figure 5-3. Full statistical analyses of experiment can be found in Extended Data Figure 5-4.

Motivated by our observations of increased sensitivity to GABAergic stimuli and previous reports of increased REM sleep in *Mbnl2* KO mice (Charizanis et al., 2012), we examined baseline sleep behavior, which is known to involve inhibitory mechanisms (Vacas et al., 2013). The video tracking software used enabled us to assess a range of parameters associated with sleep/wake behavior, in particular 40-s periods of immobility, which has been previously used as a proxy for sleep in studies that also measure sleep by EEG (Fisher et al., 2012). At baseline, *Mbnl2* KO mice (*N* = 13) traveled significantly less (*t*_(3.02)_ = 24.36, *p *=* *0.005, Welch’s *t* test) and spent a significantly longer time immobile as compared with WT littermates (*N* = 18) within a 4 h test period (*t*_(2.36)_ = 31.99, *p *=* *0.02, Welch’s *t* test; Fig. 5*A*). We also investigated these behaviors in *Mbnl2* KO and WT mice following treatment with the benzodiazepine rescue agent, flumazenil. Flumazenil has historically been used to treat benzodiazepine overdose (Sanders et al., 1991; Spivey, 1993; Kralic et al., 2002), and recently idiopathic hypersomnia (Trotti et al., 2016). We hypothesized that flumazenil might rescue, at least partially, changes in periods of immobility in *Mbnl2* KO mice. To control for variability of drug effects between animals, we used a within-subject experiment (Fig. 5*B*). A three-way repeated measures ANOVA, with genotype, treatment, and time as matching factors revealed a significant effect of time in WT mice (*F*_(3,30)_ = 13.14, *p *<* *0.0001, ANOVA). Tukey’s multiple comparison’s test revealed a significant difference in immobility between first and fourth hour of test (*F*_(3,30)_ = 13.14, *p* = 0.0018, ANOVA). Genotype, treatment, and time as matching factors revealed a significant interaction effect of flumazenil and genotype on total time immobile for *Mbnl2* KO mice across multiple time points (*F*_(1,10)_ = 6.398, *p *=* *0.029, ANOVA; Fig. 5*C*). Notably, an increase in immobility across all time points was seen in WT mice treated with flumazenil. This effect in WT mice may be related to studies showing that flumazenil can have a PAM effect (Safavynia et al., 2016). In contrast, *Mbnl2* KO mice administered flumazenil only showed a slight increase in immobility at 1 h; however, became significantly less immobile over time (*t* = 2, 3, and 4 h; Fig. 5*C*). We also found the difference of time immobile (FLZ-VEH) to be significantly decreased in *Mbnl2* KO mice (Extended Data Fig. 5-1), and analysis of the mean of all these differences (FLZ-VEH) per mouse is summarized in the table (Extended Data Fig. 5-1). WT mice showed overall increasing immobility at all time points. Individual plots of mice and their response to flumazenil are shown in Extended Data Figure 5-2. No clear correlations were found between PSI for g2L and behavioral responses among *MBNL2* KO mice (all values shown in Extended Data Figure 5-3, and full statistical analyses in Extended Data Figure 5-4).

10.1523/ENEURO.0218-22.2022.f5-1 Extended Data Figure 5-1 Flumazenil (Ro 15-1788) administration selectively decreases time immobile in *Mbnl2* KO mice versus WT mice. Differences of time immobile (FLZ-VEH) is shown for each mouse (*t*_(3)_ = 3.18, *p *=* *0.05 paired *t* test). Negative values denote decreased immobility, whereas positive values denote increased immobility. Table summarizes data shown in Figure 5*A* to indicate percentage of mice less immobile with flumazenil (Ro 15-1788) administration and mean differences in time immobile with flumazenil (Ro 15-1788) versus vehicle administration. Download Figure 5-1, TIF file.

10.1523/ENEURO.0218-22.2022.f5-2 Extended Data Figure 5-2 ***A***, Individual plots of WT mice show overall increased immobility in response to flumazenil (Ro 15-1788) compared to vehicle over 4 h of duration. ***B***, Individual plots of *Mbnl2* KO mice show overall decreased immobility in response to flumazenil (Ro 15-1788) compared to vehicle at *t* = 2, 3, 4 h. Download Figure 5-2, TIF file.

10.1523/ENEURO.0218-22.2022.f5-3 Extended Data Figure 5-3 RNA fragment analysis of Gabrg2S/L ratios confirm mis-splicing in *Mbnl2* KO mice from flumazenil (Ro 15-1788) experiment. Download Figure 5-3, TIF file.

10.1523/ENEURO.0218-22.2022.f5-4 Extended Data Figure 5-4 Statistical results from sleep and flumazenil experiment. Download Figure 5-4, XLS file.

## Discussion

Here, we take a behavioral neuropharmacology approach to uncover sensitivity to GABA in *Mbnl2* KO mice. We also validate previous transcriptomic studies of *Gabgr2L/S* ratios using two methods of mRNA detection (Fig. 1). *Mbnl2* KO mice exhibited delayed recovery following sevoflurane (Fig. 2), hypersensitivity to diazepam (Fig. 3) and zolpidem (Fig. 4), and decreased periods of immobility in response to flumazenil (Ro 15-1788; Fig. 5). Because *Mbnl2* KO mice do not display neuromuscular or locomotor deficits (Extended Data Fig. 2-2), our behavioral paradigms have direct implications for understanding sleep impairments and adverse responses to anesthesia, and perhaps provide broader implications for other CNS symptoms including anhedonia and cognitive deficits.

Our findings have important implications, providing a potential pathway for future mechanistic studies to further investigate adverse responses to anesthesia in DM1 (Butler et al., 2000), as currently only clinical case studies provide insight on these challenges. Clinical guidelines for DM1 patients undergoing general anesthesia emphasize strict monitoring to minimize adverse outcomes during the perioperative period, but the variable penetrance of DM1 may result in some DM1 patients undergoing surgery without knowing the associated risks. Volatile anesthetics like sevoflurane, preferentially target extra-synaptic GABA_A_-R to mediate tonic inhibition (Brickley and Mody, 2012; Hashemi et al., 2014), suggesting elevation in γ2S as a driver of prolonged recovery. Our behavioral data suggests *Mbnl2* KO mice have difficulty recovering from sevoflurane (Fig. 2). Because of the complicated nature of anesthetic administration in the DM1 population (Gupta, 2009), our findings in *Mbnl2* KO mice using sevoflurane highlight a potential aspect of impaired recovery from anesthesia in DM1 due the involvement of GABA. Emergence and recovery from anesthesia is a very involved process necessitating activity of various brain structures (Hemmings et al., 2019), such as the prefrontal cortex (Herrera et al., 2016; Huels et al., 2021) where γ2*S* subunits are abundantly expressed (Miralles et al., 1994).

*Mbnl2* KO mice share similarities to mice with specific knock-down of the γ2L subunit, which also had enhanced sensitivity to diazepam and zolpidem (Günther et al., 1995; Quinlan et al., 2000; Chandra et al., 2005). While we did not observe significant differences in LORR latency or duration in *Mbnl2* KO mice to diazepam (Fig. 3), our data displays a distinct response to two pharmacological drugs that target the γ2 subunit. Diazepam caused *Mbnl2* KO mice to LORR at significantly higher rates than WT mice, but not subsequent LORR metrics. In contrast, *Mbnl2* KO mice did not show LORR at significantly higher rates but did take longer to emerge from sedation from zolpidem (RORR, LORR duration). We also conducted a preliminary LORR test using the selective ligand for extrasynaptic GABA_A_-R, targeting δ-subunit containing GABA_A_-R, THIP (Extended Data Fig. 3-1; Belelli, 2005; Cope et al., 2005; Winsky-Sommerer, 2007). We did not observe any significant difference in LORR metrics between WT or *Mbnl2* KO mice. Taken together, these results both show altered drug sensitivity in the *Mbnl2* KO mouse model and the somewhat different responses may be attributed to distinct but overlapping mechanism of actions between diazepam and zolpidem involving the γ2 subunit. Benzodiazepines bind at the interface of the ɑ and γ2 subunits, and the selective imidazopyridine, ɑ1 antagonist, zolpidem, binds specifically at the ɑ1/γ2 interface (Kralic et al., 2002; Cope et al., 2004). Diazepam and zolpidem both share similar mechanisms of action with zolpidem requiring a phenylalanine in the 77th position within the γ2 subunit at the benzodiazepine binding site to increase binding affinity (Cope et al., 2004; Sumegi et al., 2012; Richter et al., 2020). While diazepam highly potentiates the γ2S subunit (Boileau et al., 2010), GABA potentiation by zolpidem only requires the presence of the γ2 subunit itself (Buhr and Sigel, 1997; Sancar et al., 2007). In addition, the ɑ1 GABA_A_-R subunit is the most abundantly expressed subunit in the brain and is predominantly expressed synaptically (Hannan et al., 2020). As such, instability of the γ2S subunit at the membrane and overall enhancement of the γ2 subunit in both synaptic and extrasynaptic compartments, could in part explain our differential findings using diazepam and zolpidem.

The γ2 subunit assists postsynaptic clustering of GABA_A_-Rs during synaptogenesis, postsynaptic localization and is critical for normal GABA_A_-R channel function and maintenance of GABA_A_-R and gephyrin at mature synapses (Schweizer, 2003; Brown et al., 2016). Alternative RNA splicing regulates the expression of two isoforms of the γ subunit (γ2S, short isoform; γ2L, long isoform). The long isoform, γ2L, has insertion of eight amino acids with a protein kinase C phosphorylation site, regulating synaptic stabilization and trafficking (Whiting et al., 1990; Meier and Grantyn, 2004). γ2S-containing GABA_A_-Rs are less stable within the synapse, potentially migrating to the extra-synaptic compartment (Lorenz-Guertin et al., 2018) to exhibit enhanced GABA transmission and reduced desensitization compared with γ2L-containing GABA_A_-Rs. γ2S is expressed early in development, whereas γ2L increases markedly in the adult brain (Wang and Burt, 1991). During postnatal brain development, MBNL-mediated binding promotes inclusion of exon-9 encoding γ2L (Weyn-Vanhentenryck et al., 2018); however, loss of MBNL results in exclusion of exon 9, possibly increasing expression of *γ2S* protein in adult brain of *Mbnl2* KO mice and human DM1 (Charizanis et al., 2012; Otero et al., 2021; Degener et al., 2022). Future work is needed to know whether there is altered localization of γ2S and γ2L proteins in DM1, as suggested by the RNA mis-splicing. As extrasynaptic GABA_A_-R are responsible for tonic inhibition, the results from our neuropharmacological approach may support the hypothesis of elevated expression of γ2S at extrasynaptic sites. While we did not observe any other changes in steady state levels for other GABA_A_-R subunit mRNAs in *Mbnl2* KO prefrontal cortex samples (Extended Data Fig. 1-1), it is also possible that other components in the excitation-inhibition balance could be contributing, such as the glutamate receptor ionotropic NMDA type subunit 1 (*Grin1*) that was mis-spliced as well in *Mbnl2* KO mice (Charizanis et al., 2012). Previous analyses of an altered excitatory system in *Mbnl2* KO mice found they exhibited impaired spatial memory on a hippocampal-dependent tasks such as the Morris water maze, and had reduced NMDA receptor-mediated synaptic potentials and LTP in the CA1 of the hippocampus (Charizanis et al., 2012).

One of the most clinically relevant symptoms in DM1 is excessive daytime sleepiness (Gorantla, 2021). Several studies conducted to understand sleep deficits in DM1 patients have reported altered sleep measured during night and day, respectively (Charizanis et al., 2012; Romigi et al., 2013; Miller, 2021). In our study, we investigated the effect of an acute, oral gavage of flumazenil on overall sleep deficits in *Mbnl2* KO mice across a 4-h time span which coincided with observed peak flumazenil brain plasma levels (data not shown). Our results reveal an enhanced immobility phenotype in *Mbnl2* KO mice which may correlate to sleep (Fig. 5*A*). This furthers previous work in *Mbnl2* KO mice where EEG recordings were conducted and found increased REM episodes (Charizanis et al., 2012). We were primarily interested in how flumazenil may effect *Mbnl2* KO mice activity because anesthesia and sleep share overlapping mechanisms (Allada, 2008) and flumazenil acts as an antagonist to anesthesia and benzodiazepines (Seelhammer et al., 2018; Benini et al., 2021) via binding to ɑ1/γ2 interface (Sharbaf Shoar, 2022). In the present study, flumazenil treatment resulted in opposing responses in WT and *Mbnl2* KO mice. The data provide further support for the model that MBNL depletion affects GABA responses. Flumazenil seems to primarily act as a negative allosteric modulator (NAM; Theadom et al., 2014) in *Mbnl2* KO mice to ameliorate phenotypes, as administration decreased periods of immobility; however, WT mice showed increases immobility in response to flumazenil (Fig. 5*C*). Flumazenil is known for its varied behavioral effects. Positive allosteric modulatory (PAM) activity of flumazenil *in vivo* has been reported in tests of social conflict, which, on treatment increased locomotor activities of timid mice and reduced aggression in aggressive mice (Uhlírová et al., 2004). Electrophysiological experiments highlight an increasingly complicated understanding of flumazenil activity *in vivo*, highlighting canonical and noncanonical mechanisms. For example, research has shown that flumazenil can regulate GABA_A_ receptor endocytosis and exocytosis, specifically decreasing surface expression of α4β2δ GABA_A_-R (Kuver and Smith, 2016). Within the first hour following flumazenil administration, *Mbnl2* KO mice showed decreased traveled distance and an increase in immobility (sleep metric) like their WT littermates. However, opposing effects were observed after 2 h and beyond. The method of oral gavage allows for flumazenil detection up to 8 h (data not shown); it remains to be determined whether observed pharmacological effects are because of PAM-mediated or NAM-mediated activities. Further work monitoring sleep over increased time spans, such as 24 h, via techniques that allow for sophisticated analysis of sleep architecture (REM vs NREM), is necessary to understand the potential long-term effects of flumazenil in *Mbnl2* KO.

Our results have potential therapeutic implications for the DM1 community, as patients with idiopathic hypersomnia have been treated with flumazenil for clinical benefit (Rye et al., 2012; Trotti et al., 2016). In a recent placebo-controlled, crossover study of 12 DM1 subjects, intravenously administered flumazenil did not lead to significant differences in Stanford Sleepiness Scale, Psychomotor Vigilance Test, or Patient and Clinician Global Impression up to 2 h after administration (Sampson et al., 2021). These findings could potentially be because of observations that flumazenil may exhibit PAM-like effects under some conditions, at least during early time points, like what we observed in our present study. Another important variable to consider, as discussed above, is that flumazenil may act via noncanonical mechanisms to dampen inhibitory transmission over longer time courses by promoting endocytosis of GABA_A_-Rs (Kuver and Smith, 2016). However, our study may also suggest that oral consumption could play an important role in ensuring more favorable pharmacokinetics for flumazenil activity in the brain. Our observation that *Gabgr2L/S* PSI values can modestly indicate time immobile in *Mbnl2* KO mice is also encouraging (Extended Data Fig. 5-3); however, future work to design custom antibodies to detect *Gabgr2L/S* protein will be critical in elucidating expression ratio at a functional level.

Our behavioral paradigms in *Mbnl2* KO mice need to be tested in DM1 mouse models expressing CUG repeats. Two such DM1 mouse models currently exist, showing impairments in excitatory synaptic transmission and associated impairments in learning and memory (Hernandez-Hernandez et al., 2013; Wang et al., 2017), which are likely relevant to CNS symptoms of cognitive impairments and intellectual disability (Urata et al., 2020; Miller et al., 2021). Recently, it was found that DMSXL mice have elevated extracellular GABA levels and tonic currents in the hippocampus. This dysfunction coincided with RNA foci accumulation in areas of the hippocampus and by the mis-splicing of candidate genes with relevant functions in neurotransmission, one of which being *Gabrg2* (Potier et al., 2022). These results further support our behavioral findings of increased GABA sensitivity in *Mbnl2* KO mice. Our results suggest targeting the GABA axis may be a potential therapeutic intervention, whether pharmacologically with GABA antagonists, or molecularly by modifying specific transcripts involved in neurotransmission with antisense oligonucleotides, as in the SMA field with the SMN2 transcript (Glascock et al., 2017; Wan and Dreyfuss, 2017). Furthermore, our research highlights the key role of MBNL2 and potentially GABRG2 as a candidate driver of altered GABA sensitivity in DM1.

## Synthesis

Reviewing Editor: Michael Michaelides, NIDA-NIH Decisions are customarily a result of the Reviewing Editor and the peer reviewers coming together and discussing their recommendations until a consensus is reached. When revisions are invited, a fact-based synthesis statement explaining their decision and outlining what is needed to prepare a revision will be listed below. The following reviewer(s) agreed to reveal their identity: NONE. Note: If this manuscript was transferred from JNeurosci and a decision was made to accept the manuscript without peer review, a brief statement to this effect will instead be what is listed below.

Reviewer 1

The authors have addressed the concerns raised in an attempt to improve the manuscript’s quality and further explain many aspects that were inadequately described or discussed in the previous version. As a consequence, the manuscript is considerably stronger, and I applaud the authors for their efforts.

I have two minor issues that I would advise the authors to consider.

The genetic background of the mice is still unclear. Is it possible to estimate the contribution of each genetic strain to the mixed background, or specify the number of backcrosses? This is an important point, since it may explain phenotypic heterogeneity in behavioural assessment and response to treatment.

"Figure 1-2” should be replaced with “Figure 1.1” in the legends of expanded data.
