# Supplementary figures and images for: Altered Behavioral Responses Show GABA Sensitivity in Muscleblind-Like 2-Deficient Mice: Implications for CNS Symptoms in Myotonic Dystrophy
Source: eNeuro. 2022 Oct 7;9(5):ENEURO.0218-22.2022. doi: 10.1523/ENEURO.0218-22.2022 (PMC9557336; doi:10.1523/ENEURO.0218-22.2022)

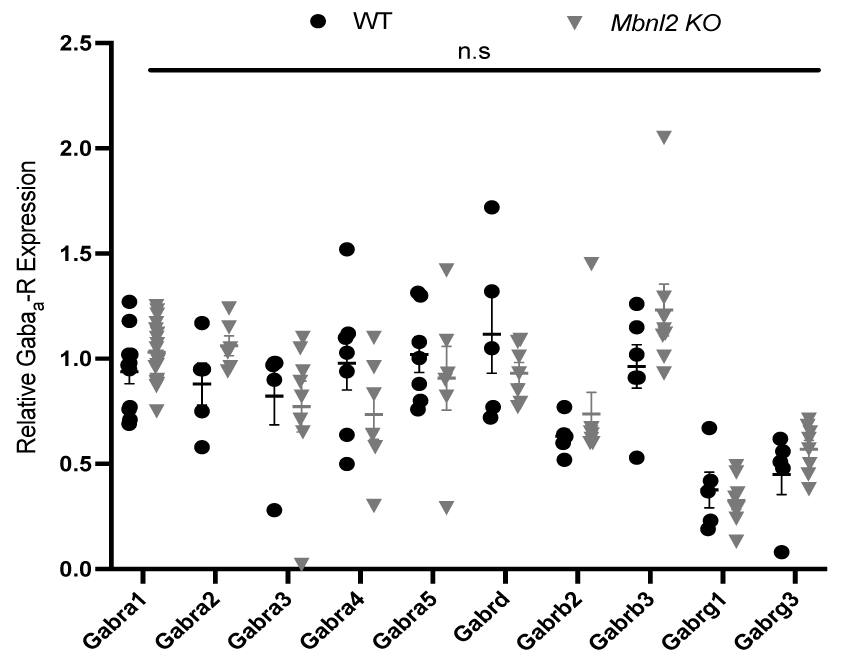

Supplement: Extended Data Figure 1-1 — Gabaa-R mRNA analysis of prefrontal cortex WT and Mbnl2 KO mice at three months of age. No significant differences detected in relative expression of majority of Gabaa mRNA receptors between Mbnl2 KO and WT mice at three months of age. A two-way mixed effect analysis revealed a significant effect between Gabaa-R (F(4.238,58.86) = 13.08, p < 0.0001, ANOVA); however, upon Sidak’s multiple comparisons test, no significant difference was detected between WT and Mbnl2 KO Gabaa-R mRNA levels (N = 7WT, 8 Mbnl2 KO), except Gabra1 (N = 11 WT, 19 Mbnl2 KO). Error bars represent SEM. Download Figure 1-1, TIF file. [file enu-eN-TNWR-0218-22-s01.tif]

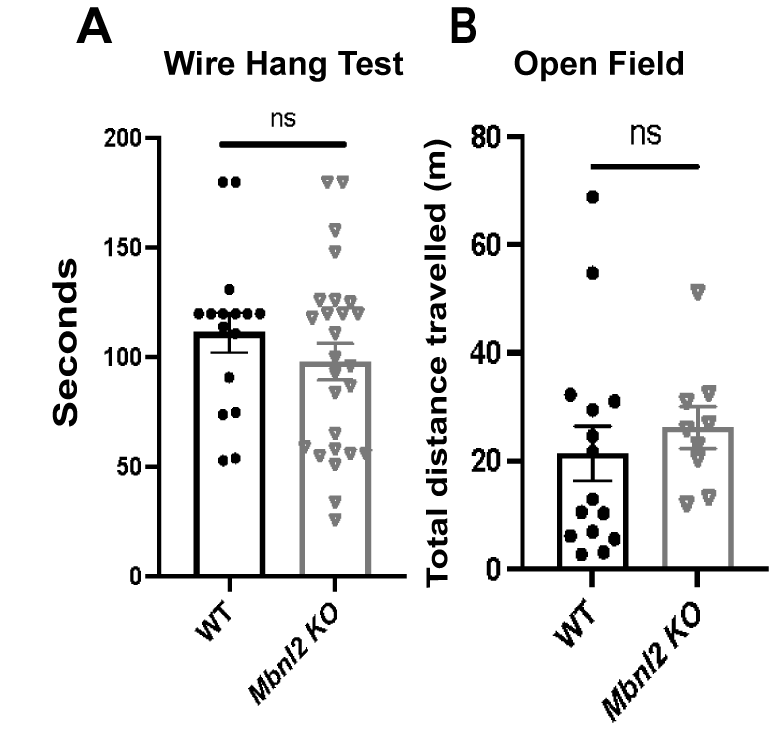

Supplement: Extended Data Figure 2-1 — No neuromuscular (Wire hang test) or locomotor deficits observed in Mbnl2 KO mice. A, Briefly, mice were placed on an elevated wire grid above which was then inverted and suspended above a cage; the latency to when the animal falls was recorded and averaged three times. No significant difference in hang time detected between genotypes (t(1.073) = 35.57, p = 0.55; Welch’s t test). B, No significant difference in distance travelled between genotypes (t(0.748) = 21.98, p = 0.46, Welch’s t test). Download Figure 2-1, TIF file. [file enu-eN-TNWR-0218-22-s03.tif]

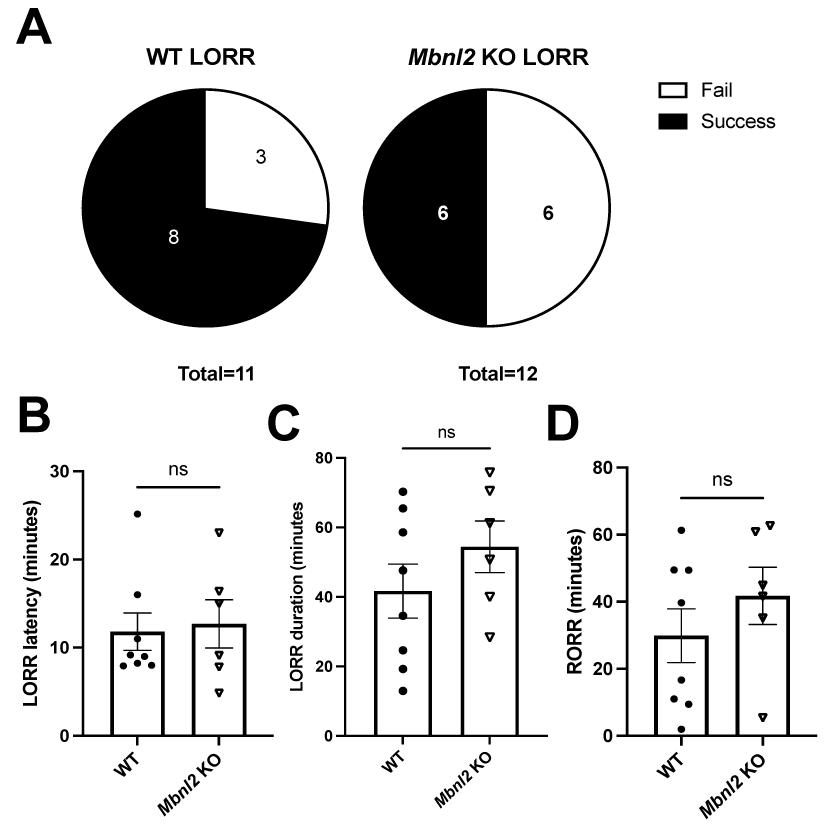

Supplement: Extended Data Figure 4-1 — No significant difference in LORR metrics between WT and Mbnl2 KO mice administered THIP. Mbnl2 and WT mice were administered 30mg/kg of THIP (IP). (A) No propensity for Mbnl2 KO (N= 6) mice to LORR at higher rate compared to WT (N=8 mice) (X2 (1, N=23) = 1.245, p= 0.26). (B-D) No significant difference in LORR latency, RORR, or LORR duration between genotypes (t(0.949)= 6.588, p = 0.38, Welch’s t-test; (t(0.793)= 11.03), p = 0. 0.39, Welch’s’ t-test, (t(0.742)=10.16, p= 0.48. Welch s t-test.) Download Figure 4-1, TIF file. [file enu-eN-TNWR-0218-22-s07.tif]

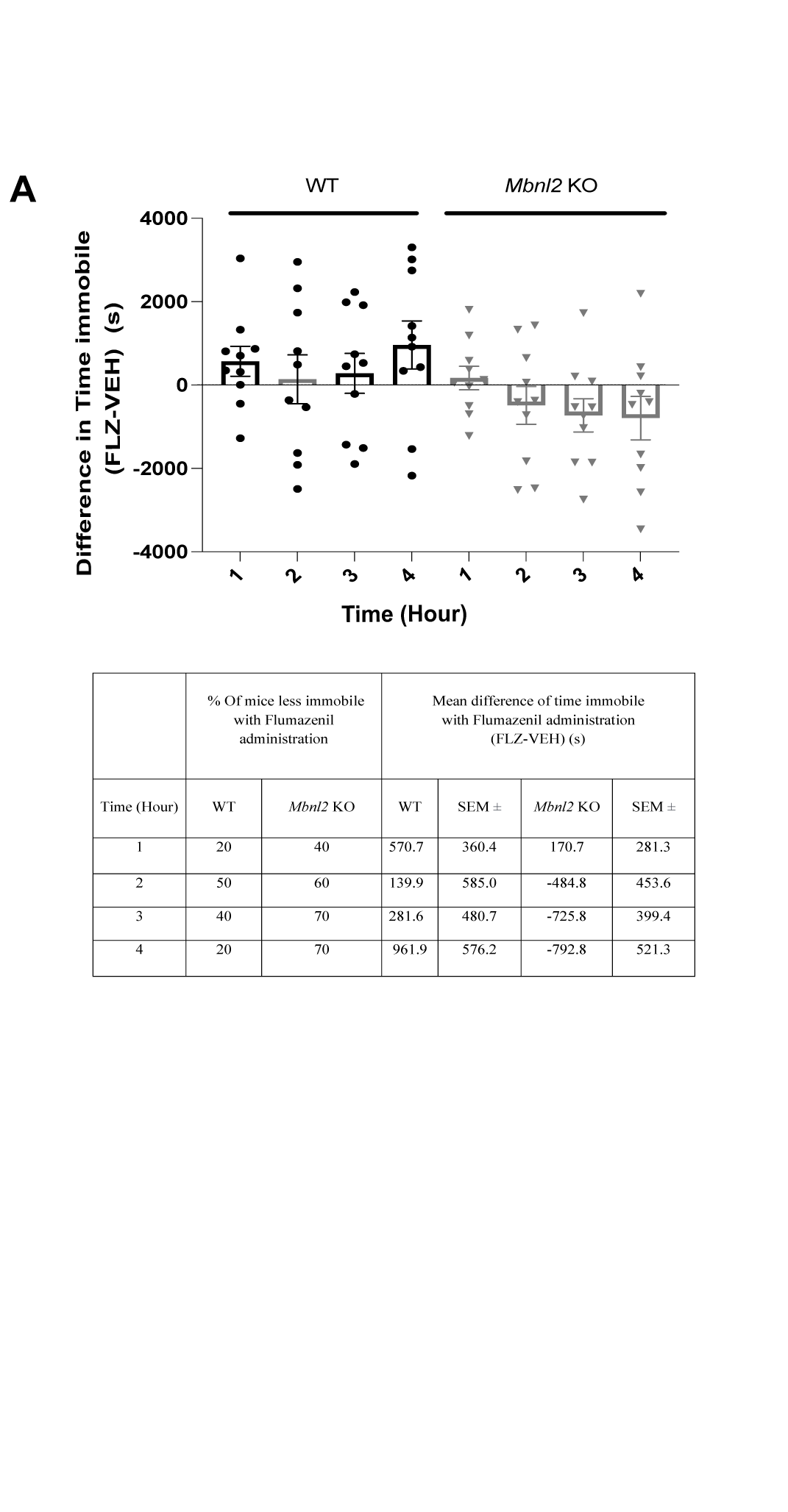

Supplement: Extended Data Figure 5-1 — Flumazenil (Ro 15-1788) administration selectively decreases time immobile in Mbnl2 KO mice versus WT mice. Differences of time immobile (FLZ-VEH) is shown for each mouse (t(3) = 3.18, p = 0.05 paired t test). Negative values denote decreased immobility, whereas positive values denote increased immobility. Table summarizes data shown in Figure 5A to indicate percentage of mice less immobile with flumazenil (Ro 15-1788) administration and mean differences in time immobile with flumazenil (Ro 15-1788) versus vehicle administration. Download Figure 5-1, TIF file. [file enu-eN-TNWR-0218-22-s10.tif]

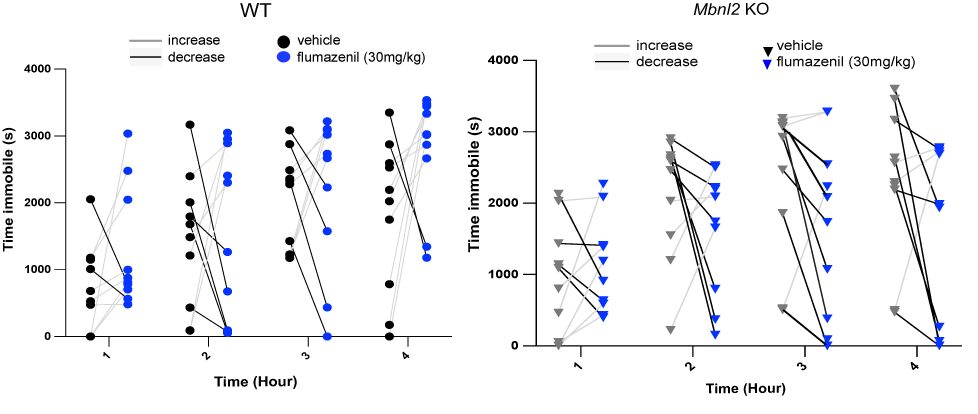

Supplement: Extended Data Figure 5-2 — A, Individual plots of WT mice show overall increased immobility in response to flumazenil (Ro 15-1788) compared to vehicle over 4 h of duration. B, Individual plots of Mbnl2 KO mice show overall decreased immobility in response to flumazenil (Ro 15-1788) compared to vehicle at t = 2, 3, 4 h. Download Figure 5-2, TIF file. [file enu-eN-TNWR-0218-22-s11.tif]

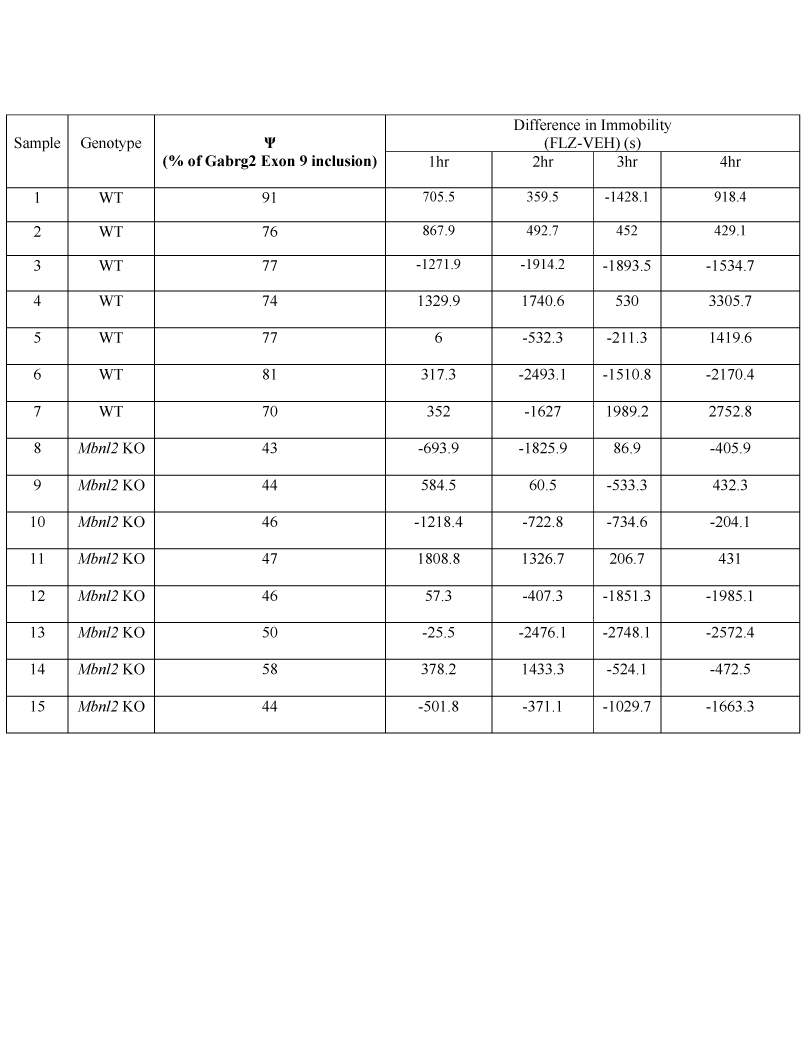

Supplement: Extended Data Figure 5-3 — RNA fragment analysis of Gabrg2S/L ratios confirm mis-splicing in Mbnl2 KO mice from flumazenil (Ro 15-1788) experiment. Download Figure 5-3, TIF file. [file enu-eN-TNWR-0218-22-s12.tif]
